# Synthetic mRNA capping

**DOI:** 10.3762/bjoc.13.274

**Published:** 2017-12-20

**Authors:** Fabian Muttach, Nils Muthmann, Andrea Rentmeister

**Affiliations:** 1 University of Münster, Department of Chemistry, Institute of Biochemistry, Wilhelm-Klemm-Str. 2, 48149 Münster, Germany; 2 Cells-in-Motion Cluster of Excellence (EXC1003-CiM), University of Münster, Germany

**Keywords:** cap analogue, cap synthesis, click chemistry, enzymatic capping, methyltransferase, RNA

## Abstract

Eukaryotic mRNA with its 5′-cap is of central importance for the cell. Many studies involving mRNA require reliable preparation and modification of 5′-capped RNAs. Depending on the length of the desired capped RNA, chemical or enzymatic preparation – or a combination of both – can be advantageous. We review state-of-the art methods and give directions for choosing the appropriate approach. We also discuss the preparation and properties of mRNAs with non-natural caps providing novel features such as improved stability or enhanced translational efficiency.

## Introduction

The 5′-cap is a hallmark of eukaryotic mRNA and involved in numerous interactions required for cellular functions. Chemically, the 5′-cap consists of an inverted 7-methylguanosine connected to the rest of the eukaryotic mRNA via a 5′–5′ triphosphate bridge. This so-called cap0 serves as quality control for correct mRNA processing and contributes to stabilization of eukaryotic mRNA [1–2], splicing [3–4], nuclear export [5], initiation of translation [6–7] and mRNA decay [8]. The most important direct interaction partners of the 5′-cap are the cap binding complex (CBC) [9–10] in the nucleus required for nuclear export and the eukaryotic translation initiation factor 4E (eIF4E) [11] in the cytoplasm which is indispensable for cap-dependent translation. Additionally, capped RNA serves as a marker for the innate immune system to distinguish triphosphorylated viral RNAs from cellular RNAs [12]. The antiviral response is among others mediated by the cytosolic receptor RIG-I which is activated by short single and double-stranded triphosphorylated RNAs and MDA-5. MDA-5 recognizes long triphosphorylated RNAs and RNAs lacking the 2′-OH methylation at the first nucleotide (cap1), a modification which is commonly observed in eukaryotes [13–15].

Besides cap0 and cap1, cap structures with further modifications exist. Additional methyl groups are often found at the second nucleotide (cap2) while in trypanosomes up to four methylated nucleotides are observed (termed cap4) [16–17].

Owing to the importance of different cap structures for recognition processes in the cell, it becomes clear that an uncapped transcript does not adequately represent a eukaryotic mRNA and that preparation of correctly capped RNAs is essential to assess the function of mRNAs in the cellular context. Furthermore, altering the cap structure bears potential to increase mRNA stability and translational efficiency – two properties which may provide the key to therapeutic applications of mRNA in the near future [18–20]. Finally, investigations of structure and mechanism of 5′-cap/protein interactions are still hampered by the difficulty of producing large quantities of homogenously capped RNA.

In this review article, we present different synthetic routes to 5′-capped mRNAs based on enzymatic, chemical or chemo-enzymatic methods. We will point out the difficulties and limitations of the different strategies and – if available – will show ways to circumvent them. This review focuses strictly on mRNA cap analogues (and some non-natural modifications); for preparation of other capped biomolecules such as capped siRNAs [21], peptidyl capped oligonucleotides [22], NAD-capped RNAs [23–24], 3'-dephospho-CoA linked RNA [25] or methylphosphate capping [26–27] we refer to the indicated articles.

## Review

### Enzymatic preparation of capped mRNA

Enzymatic preparation of capped mRNA is based on in vitro transcription (IVT) of a DNA template. While RNA synthesized via solid-phase synthesis is limited in its maximum length, RNAs with a length of several thousand nucleotides can easily be prepared through IVT. On the other hand, enzymatically produced RNA is often inhomogeneous in length and for short RNAs the yields obtained after purification may be low. This impedes the enzymatic production of short RNAs of a defined length for applications requiring defined and homogeneous RNA species. IVT produces uncapped, 5′-triphosphorylated RNA but there are two strategies to obtain mRNA with a cap, which will be discussed in detail in the following chapters.

#### Post-transcriptional capping

**Figure 1:**
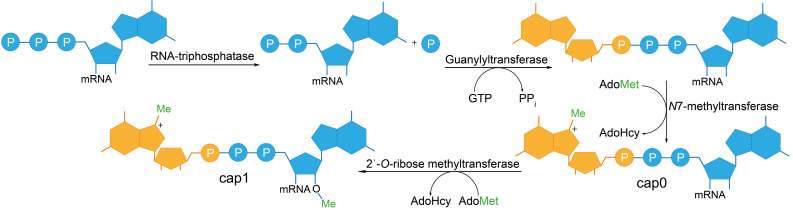
Schematic representation of enzymatic 5′-cap formation in eukaryotic mRNA. The 5′-triphosphate-end of the pre-mRNA is hydrolyzed to a diphosphate by an RNA 5′-triphosphatase. A guanylyltransferase transfers GMP onto the β-phosphate of the 5′-diphosphate to form a 5′ to 5′-triphosphate linkage. The guanine is methylated at the *N*7-position by an RNA (guanine-*N*7)methyltransferase, yielding the cap0 structure. Further methylation at the 2′-OH position of the first nucleotide results in formation of the cap1 structure.

In post-transcriptional capping, the RNA from IVT is subjected to a dedicated enzymatic capping reaction. The enzymes used in vitro originate from capping apparatuses of different eukaryotic organisms or DNA viruses and can be produced recombinantly in *E. coli* [28–29]. Enzymatic formation of cap0 comprises three consecutive reactions targeted to nascent 5′-triphosphorylated pre-mRNAs (Figure 1). First, a 5′-triphosphatase (TPase) hydrolyzes the γ-phosphate of pre-mRNA. Next, the β-phosphate of the resulting 5′-diphosphate end is coupled to GMP to form 5′–5′-linked Gppp-RNA. The responsible guanylyltransferase uses GTP as substrate and forms a covalent enzyme-(lysyl-*N*)-GMP intermediate, reminiscent of DNA ligase-AMP intermediates [30–31]. Finally, the cap structure is methylated at the *N*7-position by an RNA(guanine-*N*7)methyltransferase using *S*-adenoysl-L-methionine (AdoMet) as a cosubstrate [31].

In nature, these capping enzymes act co-transcriptionally once the transcript has reached a length of 20–30 nucleotides [32], which is enabled by their recruitment to the C-terminal domain of the RNA polymerase II [33]. In higher eukaryotes, cap1 and cap2 structures are generated by subsequent methylation of the 2′-hydroxy group of the adjacent second and third ribose, respectively [34].

These capping enzymes – e.g., from Vaccinia virus – can be harnessed for the production of capped RNA in vitro by adding them and their respective cosubstrates to the IVT reaction, as described by pioneering work of the Rosenberg group [35]. To date, the capping enzymes from the Vaccinia virus are commercially available and most widely used for post-transcriptional in vitro capping. They consist of two viral proteins D1 and D12. The triphosphatase and guanylyltransferase activity are located in the N-terminal half and the methyltransferase in the C-terminal half of the large D1 protein, whereas the small D12 protein has no catalytic activity but activates D1 [36–38].

Originally, the RNA capping with the Vaccinia capping apparatus was reported to be inefficient [35,37,39–40]. To date, the enzyme is commercially available, however, the amount of enzyme needed for the production of capped RNA in µmol scale prevents its general applicability [41]. For the application of the Vaccinia capping enzyme in the production of large-scale 5′-capped RNA, Fuchs et al. have recently reported an expression and purification protocol for the Vaccinia enzyme, allowing for capping in large quantities in a more cost-efficient manner compared to commercially available capping methods [41].

Post-transcriptional capping to obtain mRNA with a cap1 structure can be achieved using the Vaccinia mRNA cap 2′-*O*-methyltransferase which is commercially available [42–43]. Additionally, authentic mRNAs can be produced with the commercially available mScript^TM^ system which combines a T7 RNA polymerase, a trifunctional capping enzyme, a 2′-*O*-methyltransferase and a poly(A) polymerase. Albeit expensive, this system allows for production of mRNAs in one pot with claimed quantitative yields and high translational activity.

**Figure 2:**
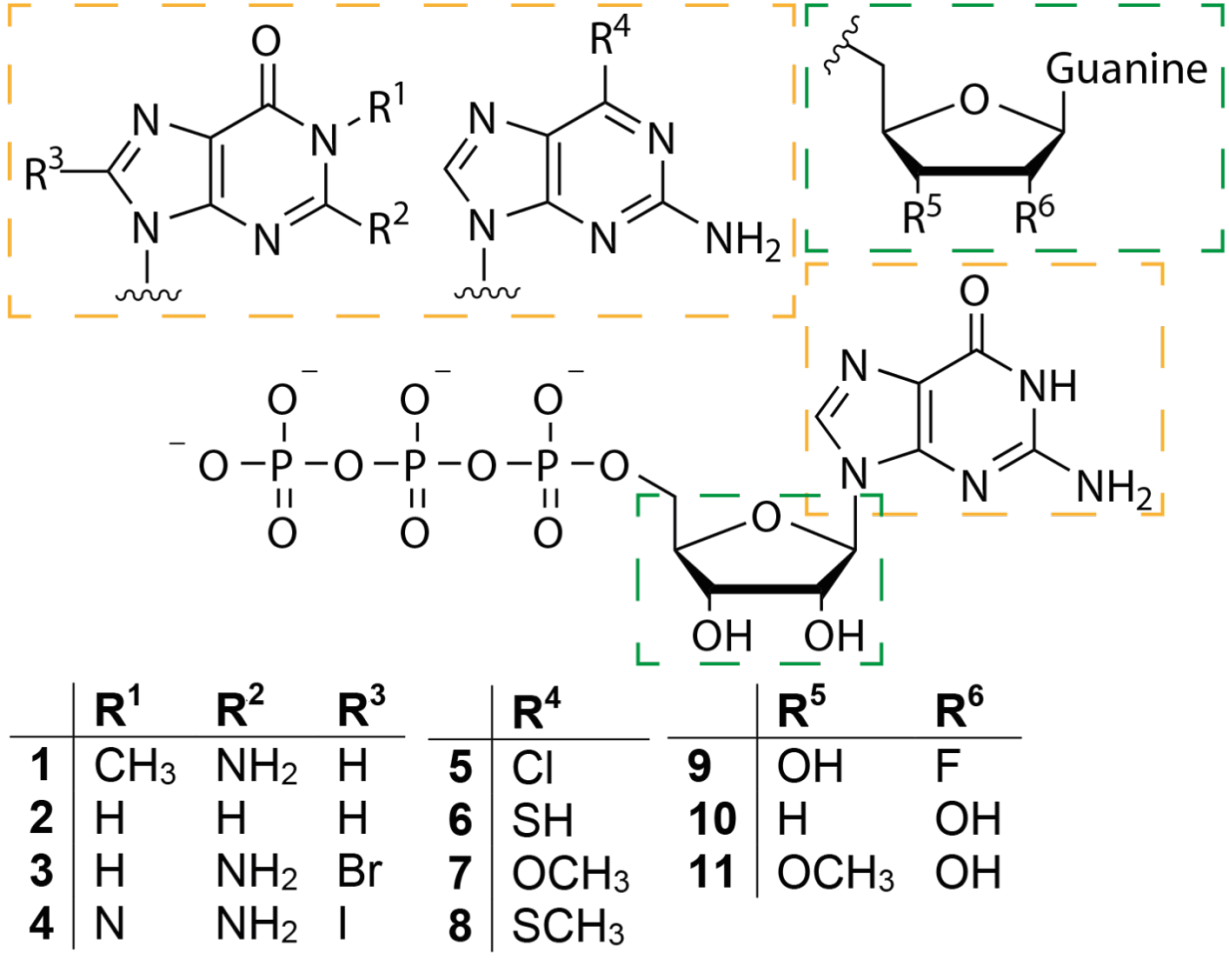
Nucleotide analogues **1**–**11** were converted by *Paramecium bursaria Chlorella virus-1* capping enzyme instead of GTP to generate RNAs with the respective caps [45].

Post-transcriptional preparation of non-natural cap analogues was achieved by capping enzymes with relaxed substrate specificity. For example, ribavirin is used as a substrate by the Vaccinia capping enzyme and can be transferred onto the diphosphate end of an RNA transcript to form a ribavirin-pppN structure. RNA transcripts blocked with ribavirin showed little translational efficiency, which might explain the antiviral activity of ribavirin [44]. Enzymatic formation of cap analogues from GTP analogues was achieved with the capping enzyme of the model organism *Paramecium bursaria Chlorella virus-1* (PBCV-1) by Bisaillon and co-workers [45]. Out of 22 nucleotide analogues tested in this study, 13 were found to form a covalent complex with the PBCV-1 guanylyltransferase (GTase) while 11 were actually transferred onto a 5′-diphosphate RNA (Figure 2). Moreover, RNAs capped with those nucleotide analogues were translated even in the absence of the *N*7-methyl group when alternative modifications enabled binding to eIF4E [45].

#### Co-transcriptional capping

**Figure 3:**
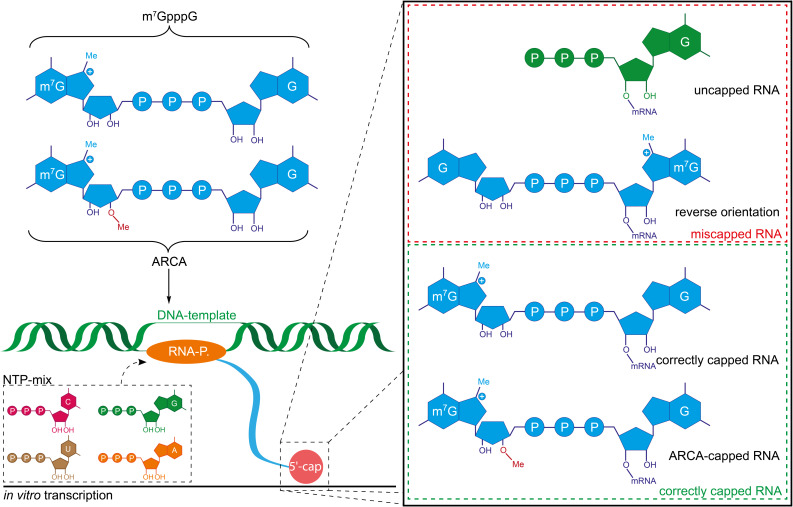
Schematic representation of co-transcriptional capping with different cap analogues. A DNA-dependent RNA polymerase initiates transcription from a DNA template by incorporation of a cap analogue, thereby producing capped RNA. When m^7^GpppG is used as a cap analogue, miscapped RNA with the cap analogue incorporated in its reverse orientation is produced in addition to the correctly capped RNA. With the ARCA cap, reverse incorporation is excluded [48].

In co-transcriptional capping, cap analogues are added directly to the IVT. Their incorporation at the 5′-end by RNA polymerases with relaxed substrate specificity (e.g., T3, T7 or SP6 RNA polymerases) directly yields the respective 5′-capped mRNA (Figure 3). Internal incorporation of cap analogues during IVT does not occur, because cap analogues lack a free 5′-triphosphate.

The most commonly used cap analogue is m^7^GpppG but several modified or alternative cap analogues are also accepted by RNA polymerases. Therefore, this route can be used to install non-natural dinucleotides at the 5′-end that are accessible for a further chemical reaction [46].

One often overlooked limitation of co-transcriptional capping is that not all mRNA obtained from IVT is capped, simply because the cap analogue competes with GTP as initiator nucleotide. Importantly, the ratio of capped/uncapped mRNA is usually not visible on a gel. This issue can be mitigated by lowering the GTP concentration or by digesting uncapped (i.e., triphosphorylated) RNA with a 5′-polyphosphatase which produces monophosphorylated RNA followed by 5′-phosphate-dependent exonuclease digestion.

Another problem encountered with m^7^GpppG as initiator is elongation into the “wrong” direction, namely at the 3′-OH of m^7^G, yielding mRNA with the cap in reverse orientation (Figure 3). Up to one half of the mRNA can contain the cap in its reverse orientation and will not be translated [47]. This problem was solved by developing anti-reverse cap analogues (ARCA) that are methylated or deoxygenated at the 3′-OH of the *N*7-methylguanosine ribose (m_2_^7,3′-^*^O^*GpppG or m^7,3′-d^GpppG). This prevents elongation at the “wrong” 3′-OH and hence ARCA caps are exclusively incorporated in the correct orientation [48–49]. Interestingly, modifications at the 2′-position of m^7^G also prevented reverse incorporation of the cap analogue [50]. The problem of orientation is circumvented when GpppA cap analogues are used in combination with the T7 class II promotor phi2.5 which allows initiating RNA synthesis with ATP. Hence, GpppA- or m^7^GpppA-capped RNAs can be produced [51]. When the common GTP-initiating T7 class III promoter phi6.5 is used, GpppA is incorporated in its reverse orientation, yielding ApppG-capped mRNAs which are biologically not active.

Due to the strict preference of bacteriophage RNA polymerases for G or A, depending on the promotor, artificial mRNAs starting with a U or C at the 5′-end cannot be prepared using in vitro transcription which limits possible applications for example in structural analysis.

#### Co-transcriptional capping of short RNA fragments

For preparation of short, capped RNA with the sequence GpppAN*_n_* or m^7^GpppAN*_n_* (1 ≤ *n* ≤ 9 nt), bacteriophage T7 gene 4 primase [52] or its active domain [53] can be used. Primase incorporates cap analogues exclusively in their correct orientation. Normally, gene 4 primase from the T7 phage produces short RNAs with the sequence pppAC from a DNA template. Matsuo et al. observed that GpppA or m^7^GpppA can be incorporated as efficiently as ATP as the first nucleotide [52]. The substrate specificity of gene 4 primase for adenosine as the first nucleotide prevents incorporation of GpppA in its reverse orientation and incorporation of GpppG altogether. This method was used for the production of isotope-labeled capped RNA for cap-eIF4E NOESY-NMR studies [52]. Peyrane et al. demonstrated that using the N-terminal fragment bearing the primase activity resulted in comparable preparation yield for the RNA while expression and solubility of the fragment were improved [53].

### mRNA cap analogues

#### Preparation of cap analogues

**Figure 4:**
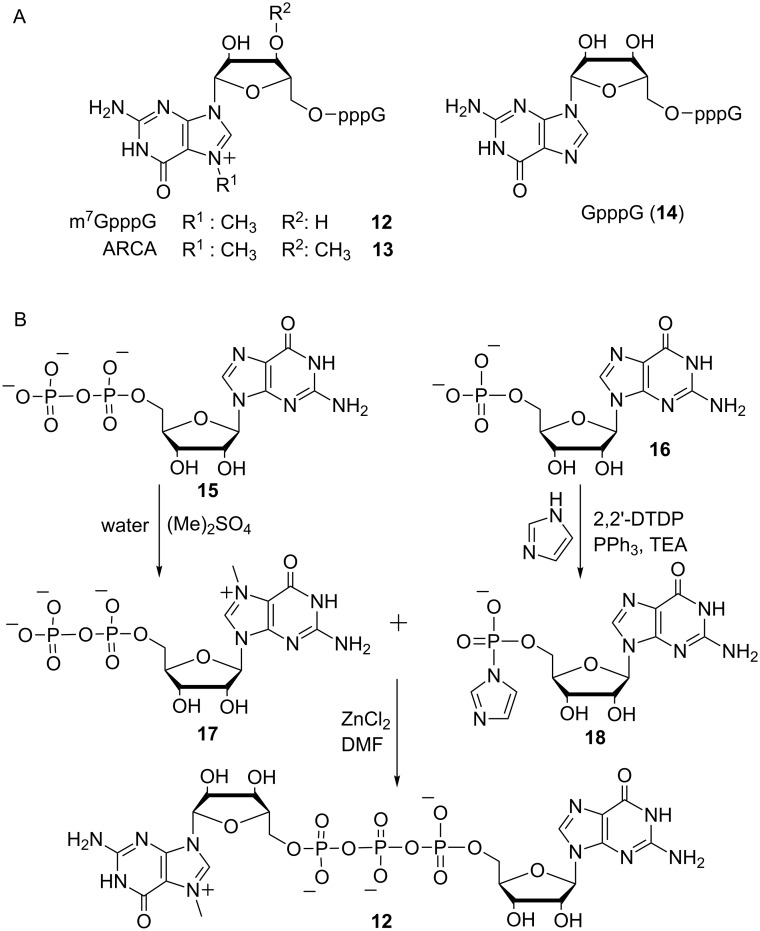
(A) Structures of commercially available mRNA cap analogues. (B) Synthetic route to cap analogues as exemplified by the synthesis of the m^7^GpppG cap analogue. 2,2′-DTDP: 2,2′-dithiodipyridine.

The co-transcriptional capping described above requires the preparation of cap analogues which are added to the transcription reaction. Ideally, these cap analogues should meet the following criteria: (i) high incorporation efficiencies when added to IVT, (ii) correct orientation when incorporated into RNA, (iii) strong binding to the cap-binding protein eIF4E, (iv) inhibitory potential when added as competitor in an in vitro translation assay and (v) high translation efficiency of resulting capped RNA. Figure 4A depicts the structure of the standard cap analogues m^7^GpppG (**12**) and GpppG (**14**). The synthesis of m^7^GpppG starts from guanosine diphosphate (GDP, **15**) and guanosine monophosphate (GMP, **16**, Figure 4B), which are both accessible by phosphorylation of guanosine [54]. Methylation of GDP gives m^7^GDP (**17**) with high yield and regioselectivity [55]. The key step in cap analogue synthesis is the formation of the triphosphate linkage. Multiple strategies have been reported which mostly rely on the same principle: One of the two nucleotides (typically the monophosphorylated nucleotide) is equipped with a good leaving group while the other one acts as a nucleophile. Different leaving groups have been exploited for the synthesis of cap analogues, comprising phenylthio [56], 5-chloro-8-quinolyl [57], morpholidate [48] and imidazolide moieties [58–59]. Imidazole activation in DMF with ZnCl_2_ was first reported by Sekine et al. [60] and is the most often used method for the formation of triphosphates. *P*-Imidazoles are known to react with numerous nucleophiles such as nucleoside mono-, -di- or -triphosphates and are typically reacted in anhydrous DMF in the presence of zinc chloride. The GMP imidazolide (**18**) is reacted with m^7^GDP (**17**) in the presence of ZnCl_2_ as catalyst to yield m^7^GpppG (**12**) [49].

In the past years, variations of this general synthetic strategy were used to obtain numerous cap analogues. Among the most interesting modifications is the above-mentioned anti-reverse cap analogue (ARCA) and GpppG which are commercially available. In addition, cap analogues with improved properties – namely binding to eIF4E, translational efficiency, and nuclease resistance – have been developed. Furthermore, cap analogues have therapeutic potential as demonstrated by a number of cap-derived translation inhibitors [61–63].

#### Applications of novel cap analogues

The search for novel – non-natural or modified – caps with improved properties has already yielded promising results. RNA capped with a locked nucleic acid (LNA)-modified dinucleotide cap analogue was translated 3-times more efficiently than regular m^7^G-capped RNA [64]. Additionally, RNA capped with the LNA cap analogue was found to be ≈1.6-fold more stable in a luciferase assay in cultured cells than the respective RNA with the standard cap. However, in this study it was not assessed how this LNA cap analogue performs in comparison to the established ARCA cap. Interestingly, a 3′-*O*-propargyl containing m^7^GpppG cap analogue also showed more than 3-fold higher translational efficiency compared to the standard cap. The cap analogue is exclusively incorporated in the correct (forward) orientation and molecular modelling studies pointed to a stronger binding of the propargyl-modified cap to eIF4E compared to the standard cap [65].

With regard to translational activity, several dinucleotide cap analogues containing a tetraphosphate were shown to be superior to the regular triphosphate in in vitro studies [66]. RNAs capped with m^7^Gp_4_m^7^G were translated with more than 3-fold higher efficiency. Interestingly, also benzyl-modified tetraphosphate cap analogues showed more than 2-fold higher translation in in vitro translation experiments. In a further step, tetraphosphates with methylene(bisphosphonate) moieties were prepared which improved binding to eIF4E and in some cases conferred enzymatic resistance against DcpS degradation [67]. *N*^2^-Triazole-containing monophosphate cap analogues were shown to be as efficient as m^7^GpppG in translation inhibition assays [68].

Further modifications can be placed in the phosphate moieties. ARCA-capped RNAs substituted with a sulphur atom at the β-position were shown to be resistant to the Dcp1/2 decapping complex from *S. pombe* while at the same time displaying high affinity to eIF4E and being translationally active when incorporated into RNA [69–70]. These properties were further improved with a range of 1,2-dithiodiphosphate cap analogues, some of which showed significantly improved stability when incorporated in an mRNA and applied in dendritic cells [71].

Furthermore, cap analogues providing additional functions were synthesized. A photo-crosslinking cap analogue containing a 6-thioguanosine was prepared which allowed for selective crosslinking [72]. Successful crosslinking was exemplified by the intrastrand crosslinking of histone H4 mRNA capped with a 6-thioguanosine cap analogue. Synthesis of biotin-labeled caps was achieved with a 2′-NH_2_-modified cap analogue which was reacted with an *N*-hydroxysuccinimide biotin active ester [73]. The biotin-labeled cap analogue could be incorporated into mRNA during IVT and retained binding to eIF4E and translational activity in an in vitro translation assay.

Besides their use in the preparation of cap-modified RNAs via IVT, cap analogues have found alternative applications. Since cap-binding proteins (e.g., eIF4E and DcpS) have high affinity to cap analogues, resins functionalized with the cap analogue m^7^GTP can be used to purify binding proteins from fractionated cell lysates [74–76]. Using m^7^G-modified sepharose resins, novel cap-binding proteins such as gemin-5 could be identified [77]. The affinity resins can be stabilized via methylene moieties, preventing enzymatic degradation of the cap analogue [78].

In recent years, cap analogues started to be recognized as inhibitors of translation by interfering with the eIF4E-RNA cap interaction. In tumorigenesis, oncogenic activity of eIF4E was attributed to its ability to activate translation [79]. Besides standard cap analogues which have long been used for eIF4E inhibition in vitro [80], the pro-drug 4Ei-1 bearing an *N*7-benzyl moiety was shown to be a potent inhibitor of cap-dependent translation in zebrafish [81]. Poor cellular uptake of cap analogues could be circumvented by coupling to an adenovirus-like particle, resulting in inhibition of hepatocellular carcinoma growth in a rat model [82]. Recently, an artificially capped RNA was prepared bearing an orthosteric eIF4E inhibitor at its 5′-end [83–84]. RNA with this cap surrogate retained binding to eIF4E as measured by surface plasmon resonance (SPR). This work provides the basis for introduction of other artificial cap analogues at the 5′-end aiming to modulate biological activity of the resulting RNAs.

#### Enzymatic modification of chemically synthesized cap analogues

An alternative to the complete chemical synthesis of cap analogues is the use of enzymes to functionalize standard cap analogues. This approach benefits from the specificity of enzymes, hence the functional moieties are directly introduced at defined positions of the mRNA cap. In the past years, our group developed chemoenzymatic strategies for modification and functionalization at the *N*7- and *N*^2^-position. Enzymatic modification is based on methyltransferases which naturally transfer a methyl group from their cosubstrate *S*-adenosyl-L-methionine (AdoMet) to the target molecule [85]. Functionalized side chains can be transferred from AdoMet analogues if an appropriate promiscuous methyltransferase is available [86]. Importantly, an unsaturated bond has to be present in β-position of the sulphonium center which stabilizes the transition state in the enzymatic transfer from the AdoMet analogue [87].

**Figure 5:**
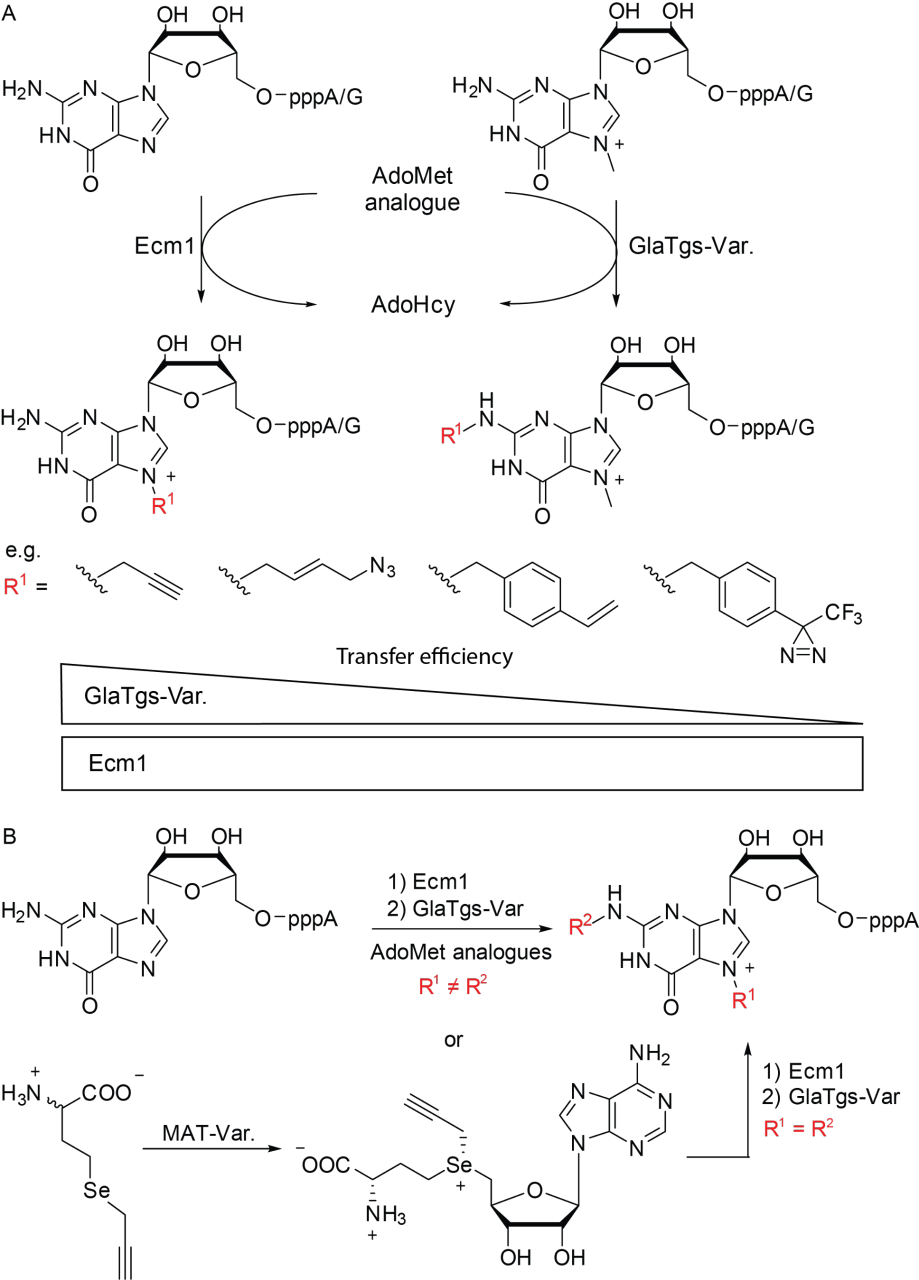
Enzymatic modification of cap analogues at their *N*^2^- or *N*7-position or a combination of both. (A) Functional moieties such as alkynes and azides can be enzymatically transferred to the *N*^2^-position using GlaTgs-Var. or *N*7-position using Ecm1 [88–91]. While transfer efficiencies decrease with increasing sterical demand when using GlaTgs-Var., transfer efficiencies are largely independent of size with Ecm1. (B) Both enzymes can be combined to yield dual or double modified cap analogues. The AdoMet analogue can also be prepared enzymatically starting from a (seleno)-methionine analogue and a MAT-Var [95].

Engineering of the trimethylguanosine synthase GlaTgs2 from the protozoan *Giardia lamblia* resulted in a variant (V34A) which accommodated AdoMet analogues with bulkier side-chains and transferred various functional moieties including propargyl, pentenynyl, azidobut-2-enyl and 4-vinylbenzyl to the *N*^2^-position of capped RNA or mRNA cap analogues such as m^7^GpppA, m^7^GpppG or m^7^GTP (Figure 5A) [88–91]. Recently, we revealed that the *N*7-cap methyltransferase Ecm1 from *Encephalitozoon cuniculi* is highly promiscuous. Sterically very demanding AdoMet analogues bearing for example a norbornene or 4-vinylbenzyl moiety were efficiently converted [92–93]. The pronounced promiscuity can be attributed to the structure of Ecm1 which forms a substrate binding cleft rather than a pocket [94].

Vinylbenzyl-modified cap analogues (bearing the modification at either the *N*7 or *N*^2^-position) provided a platform for inverse electron-demand Diels–Alder reactions with tetrazine conjugates and for photo-click reactions using tetrazoles. Even photo-crosslinking moieties were enzymatically transferred to the *N*7-position of the mRNA cap from suitable AdoMet analogues. Notably, quantitative modification at the *N*7-position was achieved [96]. Diazirine and aryl–azide photo-crosslinker moieties were functional showing cross-linking to the cap-binding protein eIF4E. Microscale thermophoresis revealed that these crosslinker-modified caps still bound to eIF4E, albeit with strongly decreased affinity. Translation was highly susceptible to modifications at the *N*7-position of the mRNA cap. While for *N*7-allyl or *N*7-azidobutenyl modifications no translational activity was observed in vitro, the *N*7-benzyl modification showed residual activity. This may be attributed to a stacking of the benzyl-moiety between tryptophans in the eIF4E binding pocket [63,66,97].

Enzymatic modification at the *N*^2^- and *N*7-position can also be combined to yield double and dual-modified cap analogues. Modification of the *N*^2^-position by Tgs-enzymes is dependent on methylation at the guanine *N*7-position, which results in a positive charge. However, GlaTgs activity relies on the positive charge rather than the methyl group itself, as exemplified by studies showing that *N*7-ethyl and *N*7-benzyl-modified cap analogues are still substrates for GlaTgs [98]. This allowed us to enzymatically prepare cap analogues with different combinations of functional moieties (Figure 5B) [95]. A 4-vinylbenzyl/azido dual modification allowed appending two different fluorescent dyes which could be applied as FRET pair. In this case, labeling was achieved in two bioorthogonal reactions, an iEDDA and a SPAAC reaction. Furthermore, dual modification with an azido and an alkyne function enabled fluorophore/biotin labeling using a combination of SPAAC and CuAAC reaction. Efficient double labeling of the mRNA cap with alkyne moieties could also be achieved based on a recently reported enzymatic cascade reaction [99]. In this system a *Se*-propargyl-modified AdoMet analogue (SeAdoYn [100]) was prepared enzymatically from the respective methionine analogue and ATP by a methionine adenosyltransferase variant (MAT-Var.). The AdoMet analogue was directly converted by the methyltransferases, resulting in double alkyne modified cap analogues [95].

### Chemical synthesis of capped mRNA

#### Solid-phase synthesis of capped RNA

Chemical synthesis of capped RNA is based on the solid-phase synthesis of RNA followed by chemical or enzymatic installation of the 5′-cap. The general principle of solid-phase RNA synthesis is beyond the scope of this review and has been described in excellent review articles [101–104]. The longest RNA synthesized via solid-phase chemistry to date has a length of 170 nucleotides and was prepared with the 2-cyanoethoxymethyl (CEM) as the 2′-OH protection group [105].

Chemical synthesis of 5′-capped RNA in solution was originally reported to be low yielding, slow (reaction times of 6–10 days), and not suitable for large-scale preparations [58,106–110]. Since then, several groups improved the chemical synthesis of capped RNA via solid-phase synthesis. The highly base-labile m^7^G moiety turned out to be a limiting factor because it is not compatible with standard solid-phase deprotection protocols. Due to its positive charge, the m^7^G moiety is hydrolytically less stable than other purine nucleosides. Under basic conditions which are commonly used for RNA deprotection and cleavage from the solid support, opening of the imidazole ring of the 7-methylguanine would occur [111]. Thus, for synthesis of the cap structure on the solid support, standard deprotection with ammonia is not possible.

**Figure 6:**
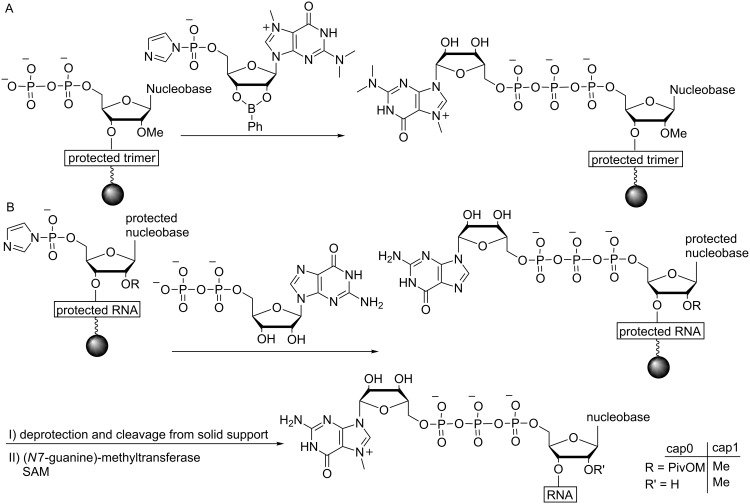
Synthesis of cap-containing RNA by solid-phase synthesis. (A) A TMG-capped mRNA was synthesized starting from an RNA tetramer which was subjected to 5′-terminal pyrophosphorylation followed by reaction with a 2,2,7-trimethylguanosine 5′-phosphorimidazolide derivative [112]. Subsequent cleavage from the solid support was achieved using 80% AcOH (rt, 24 h) and TBDMS protecting groups were removed with HCl (pH 2, rt, 12 h). (B) Large-scale production of RNAs with cap0 or cap1 by a combination of solid-phase synthesis and enzymatic methylation [111]. Deprotection conditions: DBU (1,8-diazadicyclo[5,4,0]undec-7-ene) in acetonitrile (rt, 3 min) followed by treatment with aqueous ammonia (rt, 3 h).

An early example of capped RNA prepared by solid-phase synthesis was reported by the group of Sekine in 2001 [112]. A 2,2,7-trimethylguanosine (TMG)-capped trinucleotide block of U1 snRNA with the structure m_3_^2,2,7^G^5′^pppAm^2′^Um^2′^A was prepared, starting from a 5′-phosphorylated trimer synthesized by standard phosphoramidite chemistry. To address the problem of m^7^G instability under basic conditions, the TMG-capping reaction was carried out upon deprotection of all base-labile groups. Utilization of a novel, acid labile linker to the solid support allowed for subsequent release of the RNA. However, due to overall low coupling efficiencies and isolated yields (the compound was isolated in 20% overall yield after anion-exchange chromatography), this method was not used for large scale synthesis of capped RNA (Figure 6A). As the low reaction yields are mainly caused by the multistep preparation of the triphosphate bridge, the Sekine group presented a synthetic route to RNA bearing a 5′-terminal TMG-capped pyrophosphate linkage on solid support. Since pyrophosphate formation is easier than triphosphate formation, this route resulted in higher coupling yields. Whether this RNA is still biologically active remains to be demonstrated [113]. Furthermore, these capping approaches can be used to produce biologically relevant RNA. U1snRNA was prepared via enzymatic ligation of a short RNA (10 nt long) containing a trimethylated m_3_^2,2,7^G cap moiety to a 154 nt long RNA produced via IVT. The respective U1snRNAs with a pyrophosphate bridged TMG cap and a TMG cap containing an ethylene glycol linkage were also produced [114].

Unlike IVT, solid-phase synthesis offers the flexibility to introduce modified nucleotides at specific positions. Chemical synthesis of the intricate trypanosomatid cap4 structure, characterized by 2′-*O*-methylation of the first four nucleotides and additional methylation at the first adenosine and the fourth uridine, was reported in 2004 by the group of Darzynkiewicz. The preparation was achieved by reacting an imidazole activated m^7^GDP with the 5′-phosphorylated tetramer [115]. This cap was successfully used for affinity purification of trypanosomatid cap4 interacting proteins [116–117].

Nagata et al. reported on the first preparation of mature mRNA based on a chemically synthesized RNA strand which was shown to be biologically active in cells [105]. This was achieved by combining solid-phase synthesis and enzymatic modification. Specifically, 5′-diphosphorylated RNAs (up to 170 nt long) were chemically synthesized, cleaved from the solid support, deprotected and purified. This was followed by enzymatic capping, 2′-*O*-methylation and polyadenylation.

A combination of chemical synthesis and enzymatic modification was also used by Thillier et al. for the large scale synthesis of capped RNA. Herein, to circumvent the problem of m^7^G instability, non-methylated capped RNAs were first synthesized using the phosphoramidite 2′-*O*-pivaloyloxymethyl method, followed by enzymatic *N*7 methylation using the human (guanine-*N*7)-methyltransferase (Figure 6B). A cap1 structure could also be obtained via 2′-OH methylation of the terminal nucleotide [111]. This approach was applied in collaboration with other groups for the production and investigation of capped RNA [118].

In summary novel chemical capping strategies enable preparation of capped RNAs in high yield and independent of the sequence, providing access to RNAs that could not be prepared via IVT. However, preparation of biologically relevant mRNAs that are typically thousands of nucleotides long is not directly feasible, as the longest chemically prepared RNA to date comprises 170 nt. Methods combining chemical and enzymatic preparation of capped RNA bear potential to resolve these limitations and will be described in the following.

#### Combining chemical and enzymatic methods: primer extension

Engineering of the replicative DNA polymerase from *Thermococcus gorgonarius* (Tgo) into a DNA-dependent RNA polymerase (termed TGK) enabled production of up to 1,700 nt long RNAs from a ssDNA template and an RNA primer [119]. The primer-dependent RNA synthesis obviates the need to initiate RNA synthesis with pppG in contrast to most other RNA polymerases used for conventional IVT. TGK turned out to accept a number of variations at the 5′-end including an oligoribonucleotide primer containing the desired cap. This approach unites the flexibility of RNA synthesis and processivity of RNA polymerases for the preparation of long and cap modified RNAs. Using this system, several biologically relevant RNAs such as GFP RNA, firefly luciferase RNA and m^7^Gpppm^6^A_m_-RNA were produced [118–119].

#### Click chemistry for the preparation of capped RNAs and cap analogues

**Figure 7:**
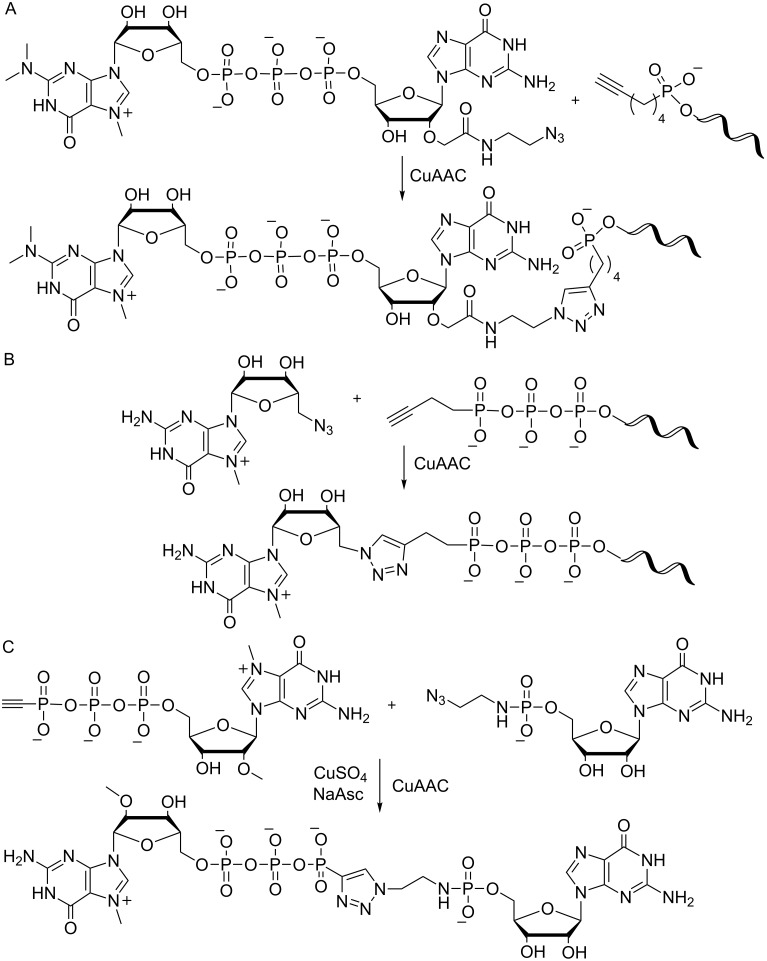
Click chemistry for the preparation of capped RNA and cap analogues. (A) Preparation of capped RNA via a copper-catalyzed azide–alkyne cycloaddition (CuAAC) of an azido-modified cap analogue with a 5'-alkyne bearing RNA [120]. (B) An alkyne-modified triphosphorylated RNA is reacted with 5'-azido-methylguanosine in a CuAAC [121]. (C) Alkyne- and azido-containing nucleotide building blocks are reacted in a CuAAC to give a functional cap analogue [121].

As an alternative to preparation of longer RNA via IVT, different hypermethylated cap analogues with a 2′-azido moiety allowed for reaction with an alkyne-modified RNA in a CuAAC reaction to yield cap modified RNA – albeit with a non-natural linkage (Figure 7A) [120]. This capping strategy also worked with an alkyne-modified triphosphorylated RNA and 5′-azido modified methylguanosine resulting in a capped RNA containing a triazole linkage after CuAAC reaction (Figure 7B) [121]. In a similar approach a 5′-azido-modified RNA was prepared by solid-phase synthesis and reacted with an alkyne-functionalized m^7^G-cap analogue in a CuAAC reaction [122]. Besides its utility on long RNA, this click chemistry approach was also applied to the chemical synthesis of cap analogues, simplifying the typically laborious and time-consuming synthesis [121]. A plethora of cap analogues was synthesized replacing one phosphate bridge with a triazole linkage. Depending on their structure and the exact positioning of the triazole linkage, modified cap analogues varied largely with regard to their functionality in in vitro translational assays, binding affinity to eIF4E and resistance to the decapping enzyme DcpS. Best translational efficiencies similar to the standard cap were achieved with a tetraphosphate cap analogue containing a triazole bridge (Figure 7C).

## Conclusion

The 5′-cap is the key modification of eukaryotic mRNAs and provides an interaction platform for proteins involved in fundamental processes like nuclear export and translation. Therefore, preparation of mRNAs with the canonical cap structure is indispensable for a comprehensive understanding of mRNA functions that go beyond the genetically encoded information, e.g., studies elucidating RNA-protein interactions [123] or structure analysis [124]. Moreover, artificially capped RNAs or RNAs with modified 5′-caps may provide a means to control or selectively block some of these functions, resulting in improved translational efficiency or higher stability.

Depending on the desired length of the capped RNA fully synthetic, enzymatic or a combination of both strategies is feasible and allows production of differently capped RNAs with a length ranging from several nucleotides to authentic mRNAs (>1000 nt). Novel strategies for the synthesis of cap analogues have led to the development of 5′-caps with tailored functionalities which are, for instance, resistant to enzymatic degradation or bear functional moieties for additional bioconjugation reactions. A combination of chemical 5′-cap analogue synthesis followed by enzymatic modifications has further allowed conferring novel functionalities (e.g., photo-crosslinking moieties) which were previously not easily accessible. Combining enzymatic modification at different positions (e.g., *N*^2^ and *N*7-position) renders dual and double modifications possible, further diversifying mRNA 5′-cap modifications and leading to the highly regiospecific introduction of two different functionalities. Most recent developments focused on the development of completely artificial mRNA caps which conferred specific properties such as eIF4E binding and turned non-modified RNAs into strongly eIF4E-binding RNAs.
